# Radiosurgery or hypofractionated stereotactic radiotherapy after craniospinal irradiation in children and adults with medulloblastoma and ependymoma

**DOI:** 10.1007/s00381-018-4010-8

**Published:** 2018-12-04

**Authors:** Aleksandra Napieralska, Iwona Brąclik, Michał Radwan, Marek Mandera, Sławomir Blamek

**Affiliations:** 1 Department of Radiotherapy, The Maria Sklodowska-Curie Institute — Oncology Center, Gliwice Branch, Ul. Wybrzeże AK 15, 44-101 Gliwice, Poland; 2 Department of Radiotherapy and Brachytherapy Planning, The Maria Sklodowska-Curie Institute — Oncology Center, Gliwice Branch, ul. Wybrzeze AK 15, Gliwice, 44-101 Polska; 3 Department of Pediatric Neurosurgery, Medical University of Silesia, Katowice, Poland

**Keywords:** Radiotherapy, CSI, Recurrence, Reirradiation

## Abstract

**Purpose:**

To assess the results and tolerance of radiosurgery/hypofractionated stereotactic radiotherapy performed after craniospinal irradiation for recurrent tumor.

**Methods:**

Fourteen patients aged 3–46 years, diagnosed with medulloblastoma (10), anaplastic ependymoma (3), and primitive neuroectodermal tumor (1). All patients had craniospinal irradiation (CSI) with the total dose of 30.6–36 Gy and boost to 53.9–60 Gy either during primary or during second-line treatment. Twelve patients were irradiated with a single dose of 6–15 Gy (median 14.5 Gy). One received three fractions of 5 Gy and one six fractions of 5 Gy. In statistical analysis, the Kaplan-Meier method and log-rank test were used. The overall survival was calculated from the date of the end of stereotactic radiosurgery to the date of death or last contact.

**Results:**

Recurrences were diagnosed after the median time of 16 months after the end of primary treatment. Eleven patients died during the follow-up. The follow-up for the 3 patients still alive was 6.7, 40.5, and 41.4 months, respectively. One- and 2-year overall survival (OS) was 70% and 39%. Patients who had ECOG performance status of 0 at the time of diagnosis of the disease trended to have better 2-year OS compared to those evaluated as ECOG 1 (*p* = 0.057). Treatment results were evaluable in 12 patients. Local control (stabilization or regression of the lesion) was achieved in 9 (75%). Overall disease progression was 67%. No patient developed radiation-induced necrosis. The treatment was well tolerated and no serious adverse effects were observed. Eleven patients were given steroids as a prevention of brain edema and four of them needed continuation of this treatment afterwards. In 7 patients, symptoms of brain edema were observed during the first weeks after reirradiation.

**Conclusions:**

Stereotactic radiosurgery or hypofractionated stereotactic radiotherapy is an effective treatment method of the local recurrence after CSI and can be performed safely in heavily pre-treated patients.

## Introduction

Medulloblastoma is one of the most common primary brain tumors in children and one of the rarest in adults [1, 2]. In the recent years, the results of the treatment have significantly improved by the use of combined therapy–surgery, craniospinal irradiation with dose escalation to the tumor bed or residual tumor, and, in children, chemotherapy [1–4]. The role of chemotherapy in the first-line treatment in adults is less clear due to increased risk of toxicity of systemic treatment. Recent studies of Rare Cancer Network Group showed that this group of patients may also benefit from systemic therapy [5]. But still, in this group it is used less frequently, usually in selected high-risk patients [2, 6]. Five-year progression-free survival (PFS) in standard-risk patients is within the range of 57 to 82%, and the 5-year overall survival (OS) varies between 80 and 87%, both in children and in young adult patients [1–6].

Despite the advances in treatment of patients with medulloblastoma and anaplastic ependymoma, there are still no clear guidelines concerning treatment of recurrence. The outcome is poor, irrespective of the implemented treatment methods (surgery, chemotherapy, combined in some cases with bone marrow transplantation, brachytherapy, radiosurgery, or hypofractionated stereotactic radiotherapy). So far, only two authors reported 5-year OS of 55–65% [7–25].

Publications on the reirradiation of patients after radiotherapy of cerebrospinal axis are scarce, and radiotherapy is often not considered a salvage treatment because of its potential toxicity (including radiation necrosis of the brain), young age of patients, and uncertain effectiveness [5, 10–25].

We present a series of patients reirradiated in a single center and evaluate the treatment results and tolerance of radiosurgery (SRS)/hypofractionated stereotactic radiotherapy (SRT) implemented after craniospinal irradiation (CSI) in patients with a recurrent tumor.

## Material/methods

### Group characteristics

Study inclusion criteria were as follows: the diagnosis of a central nervous tumor, CSI as part of initial or second-line treatment, and treatment with SRS/SRT implemented after CSI. Fourteen consecutive patients (8 females, 6 males, median age at diagnosis of the disease was 22 years), diagnosed with medulloblastoma (MB, 10 patients), anaplastic ependymoma (AE, 3 patients), and primitive neuroectodermal tumor (PNET, 1 patient), met the inclusion criteria and were enrolled into the analysis. All patients received SRS or SRT as part of the treatment of recurrence after CSI. Information on patients and treatment details was collected retrospectively from patients’ charts and treatment planning system archives.

### Primary treatment

All patients were treated with radical intent. Before and during the primary treatment, all patients were in good performance status (ECOG 0–50%, ECOG 1–50% of patients). Surgery was the primary treatment modality in all of them (in 63%, gross total resection was performed, and in 3, partial resection). Primary tumor was located in posterior cranial fossa in 10 patients (MB patients); in 3, in frontal lobe; and in 1, in temporal lobe. Six patients (43%) received adjuvant chemotherapy (5 with medulloblastoma, one with PNET). In 12 patients, CSI was a part of the primary treatment. Patients were irradiated with fraction dose of 1.5 to 1.8 Gy (median spinal fraction dose of 1.5 Gy, median cranial fraction dose of 1.8 Gy) to deliver the total dose of 30.6–36 Gy (median 36 Gy) with boost to the tumor/tumor bed up to 53.9–60 Gy (median 54 Gy). Additional unplanned surgery was performed in one patient after CSI due to aggravation of neurological symptoms. After the treatment, all patients were followed up in regional oncology centers. Patients had checkup visits every 3 to 6 months during the first 2 years after the primary treatment and every 6 months during the next years. Imaging was performed with similar consistency. Additional visits or imaging was performed when patient presented symptoms of tumor progression.

The details of the primary treatment employed in particular patients are presented in Table 1.

**Table 1:** Primary treatment in particular patients

Patient	Histopathology	Primary tumor location	Age	Surgery	CTH	CSI	Total dose
1	MB	Posterior cranial fossa	3	Non-radical surgery	Yes	No	–
2	MB	Posterior cranial fossa	4	Radical surgery	Yes	Yes	36 Gy/craniospinal axis, 54 Gy/tumor bed boost
3	MB	Posterior cranial fossa	9	Non-radical surgery	Yes	Yes	35.1 Gy/craniospinal axis, 55.1 Gy/tumor boost
4	MB	Posterior cranial fossa	10	Non-radical surgery	Yes	Yes	35.1 Gy/craniospinal axis, 55.1 Gy/tumor boost
5	MB	Posterior cranial fossa	22	Non-radical surgery	–	Yes	36 Gy/craniospinal axis, 60 Gy/tumor boost
6	MB	Posterior cranial fossa	30	Radical surgery	–	No	–
7	MB	Posterior cranial fossa	31	Radical surgery	–	Yes	36 Gy/craniospinal axis, 54 Gy/tumor bed boost
8	MB	Posterior cranial fossa	46	Non-radical surgery	–	Yes	30.6 Gy/craniospinal axis, 53.9 Gy/tumor boost
9	AE	Frontal lobe	19	Radical surgery	–	Yes	36 Gy/craniospinal axis, 60 Gy/tumor bed boost
10	AE	Frontal lobe	34	Radical surgery	–	Yes	36 Gy/craniospinal axis, 60 Gy/tumor bed boost
11	PNET	Frontal lobe	32	Non-radical surgery	Yes	Yes	36 Gy/craniospinal axis, 54 Gy/tumor boost
12	AE	Temporal lobe	22	Non-radical surgery	–	Yes	36 Gy/craniospinal axis, 60 Gy/tumor boost
13	MB	Posterior cranial fossa	33	Radical surgery	Yes	Yes	36 Gy/craniospinal axis, 54 Gy/tumor bed boost
14	MB	Posterior cranial fossa	20	Radical surgery	–	Yes	36 Gy/craniospinal axis, 54 Gy/tumor bed boost

*AE* anaplastic ependymoma, *CSI* craniospinal irradiation, *CTH* chemotherapy, *MB* medulloblastoma, *PNET* primitive neuroectodermal tumor, *RTH* radiotherapy

### Recurrence of the disease

Recurrence of the disease occurred after the median time of 16 months (range 3 to 78 months) after the end of primary treatment. In all patients, diagnosis of the recurrence was based on MRI. The most common location of the recurrent tumor was primary site. Recurrent tumor was located in posterior cranial fossa in postoperative bed in 5 patients; in 5, in frontal lobe (in one of them in postoperative bed); in 2, in temporal lobe (in postoperative bed in one); and in 2, close to the postoperative bed in parietal lobe and cribriform plate. Three patients were diagnosed with dissemination of the tumor (one in spinal canal), but all of them received chemotherapy afterwards and at the time of radiosurgery, no disease apart from the treated lesions was observed (MRI of craniospinal axis was performed to confirm that). One patient was treated for two lesions (both in the postoperative bed, close to each other, of 1.54 cc and 0.76 cc volume).

The majority of patients (93%) were in good general condition at the time of diagnosis of the recurrence (ECOG 0 or 1), and 42% of them had no clinical symptoms of the recurrence. Headaches were the most commonly reported symptom (29%). In 5 cases (36%), resection of the recurrent tumor was performed. Six patients received systemic therapy. In two patients, conventional radiotherapy with fraction dose of 2 Gy to total dose of 20 (10 fractions) and 30 Gy (15 fractions), respectively, was delivered. In one of them, with AE, the surgical cavity after resection of the recurrent tumor was irradiated with 30 Gy. During the follow-up, a small lesion in the parietal region was found. Patient was referred to a neurosurgeon who did not decide to perform another surgery. Due to small size of the lesion (less than 2 cm), the interdisciplinary board decided to treat the patient with SRS. The other patient, with PNET, was diagnosed with recurrence of the tumor in the ethmoid and nasal cavity. Due to large volume of the recurrent tumor, the first radiotherapy was delivered with conventional fractionation. Very good response to the first radiotherapy (regression of the irradiated lesion) led to the decision to additionally perform SRS. Another two patients received CSI at the time of recurrence (they did not receive that treatment before) after which they were qualified to radiosurgical treatment.

### Radiosurgery

All patients received SRS or SRT as part of the treatment of the recurrence (two after CSI, as a boost on the recurrence site). Time between CSI and SRS/SRT of recurrence ranged from 1.1 to 75.6 months (median 6.2 months). During SRS/SRT, all patients were immobilized with individualized thermoplastic masks covering head and shoulder region. Masks were fixed to the treatment couch during the treatment delivery in order to reduce patient’s motion. Treatment planning was done with the BrainLab software and pencil beam optimization algorithm was used. All patients were treated with conventional linear accelerators equipped with a micro-multileaf collimator. Radiation was delivered with conformal beam (10 patients) or intensity-modulated radiosurgery (IMRS; 4 patients) technique with 5 to 12 fields. The dose was normalized at the isocenter and planned to cover 98% of the target volume with 95% of the prescribed dose. Six to 20 MV photons were used. Twelve patients received a single dose of 6–15 Gy (median 14.5 Gy). One patient received three fractions of 5 Gy and one six fractions of 5 Gy. The youngest two patients (5 and 6 years old at the time of SRS) received short general anesthetic for the time of treatment delivery.

In all cases, recurrence was diagnosed based on MRI and clinicians engaged in the treatment of patients were able to use MRI to aid treatment planning. In all patients, the first radiotherapy treatment plans were reviewed in order to evaluate doses delivered to critical structures. This data was taken into account during the second course of irradiation. In 13 of 14 patients (including the four after resection), a recurrent tumor was visible on MRI and gross tumor volume (GTV) was defined as the contrast-enhancing lesion in T1-weighted images. In one patient, total resection of the recurrent tumor was performed and irradiated region covered the postoperative bed. There was no margin added and the planning target volume (PTV) was in fact GTV except the one patient mentioned above. PTV ranged from 0.54 to 27.04 cc (median 1.36, mean 6.42). In case of hypofractionated stereotactic radiotherapy, the target volume definition did not differ from that used for single fraction treatment.

Treatment of the recurrence in particular patients is presented in Table 2.

**Table 2:** Treatment of the recurrence in particular patients

Patient	Time to recurrence (months)	Location of recurrence	ECOG at recurrence	Surg.	CTH	RT*	TD RT (Gy)	SRS/SRT TD (Gy)	Treatment effect	Follow-up (months)	Last control status
1	10.1	Temporal lobe, out-of-field of initial RT boost	1	–	Yes (3 types)	Yes	36 Gy on craniospinal axis + 54 Gy tumor boost	10.0	CR	13.3	Dead
2	6.2	Frontal lobe, out-of-field of initial RT boost	1	–	Yes (1 type)	–		15.0	SD	15.2	Dead
3	31.4	Frontal lobe, out-of-field of initial RT boost	1	–	Yes (4 types)	–		15.0	N/A	3.8	Dead
4	17.3	Frontal lobe, out-of-field of initial RT boost	1	–	Yes (3 types)	–		8.0	N/A	13.0	Dead
5	28.5	Posterior cranial fossa, in-field of initial RT boost	0	–	–	–		14.0	CR	77.1	Dead
6	10.5	Posterior cranial fossa, in-field of initial RT boost	1	Yes	–	Yes	36 Gy on craniospinal axis + 54 Gy tumor boost	10.0	SD	26.5	Dead
7	3.4	Posterior cranial fossa, in-field of initial RT boost	0	–	–	–		12.0	CR	41.4	Alive
8	16.0	Frontal lobe, out-of-field of initial RT boost	1	–	–	–		15.0	SD	7.7	Dead
9	34.2	Frontal lobe, in-field of initial RT boost	0	Yes	–	–		15.0	PD	40.5	Alive
10	27.9	Parietal lobe, close to the field of initial RT boost	1	Yes	–	Yes	30.0 in 2 Gy fractions	15.0	SD	30.0	Dead
11	4.9	Cribriform plate, close to the field of initial RT boost	2	–	Yes (2 types)	Yes	20.0 in 2 Gy fractions	6.0	SD	17.4	Dead
12	10.0	Temporal lobe, in-field of initial RT boost	0	Yes	–	–		15.0	PD	11.4	Dead
13	8.9	Posterior cranial fossa, in-field of initial RT boost	0	Yes	Yes (1 type)	–		15.0/3 fx	PD	2.9	Dead
14	77.7	Posterior cranial fossa, in-field of initial RT boost	0	–	–	–		30.0/6 fx	CR	6.7	Alive

*CR* complete regression, *CTH* chemotherapy, *ECOG* performance status, *fx* fraction, *N/A* data not available, *SD* stable disease, *SRS* radiosurgery, *PD* progression of the disease, *RT* conventional radiotherapy, *Surg.* surgery, *TD* total dose

*RT—four patients received conventionally fractionated radiotherapy as part of the treatment of recurrence (two of them, who were not irradiated previously, CSI) before SRS/SRT

After the radiosurgery, 12 patients were systematically followed up (2 did not come for planned checkup visit). Patients had checkup visits every 3 to 6 months during the first 2 years after the SRS/SRT and every 6 months during the next years. Imaging was performed with similar consistency. Additional visits or imaging was performed when patient presented symptoms of tumor progression. In all of them, treatment effect was assessed with diagnostic imaging (all patients had MRI and some of them CT but none of them had CT as a sole follow-up modality).

### Statistical analysis

Statistica 12.0 was used for statistical analysis. The Kaplan-Meier method and log-rank test were used in statistical analysis. *p* value of less than 0.05 was considered statistically significant. Follow-up was calculated from the date of the end of radiosurgery/hypofractionated stereotactic radiotherapy of the recurrent tumor to the date of death or (in case of alive patients) last contact. Progression was defined as the occurrence of a new lesion or progression of the irradiated tumor based on MRI imaging, and the date of MRI was defined as the date of progression. Progression-free survival was defined as a period of time without tumor progression or death. Data on the date of death were obtained from the National Cancer Registry.

## Results

Local control (stabilization or regression of the lesion) was achieved in 9 patients (75%)—in 4, complete regression, and in 5 patients, the size of the irradiated tumor was stable. Progression of the irradiated lesions was observed in 3 patients. Radiation-induced changes of surrounding healthy brain tissues (edema, vascular changes) were observed in all patients, but none of them developed radiation necrosis.

The treatment was well tolerated and no serious adverse effects were observed. Eleven patients were given steroids during hospitalization for SRS/SRT as a prevention of brain edema. Among them, four needed continuation of this treatment afterwards. In seven patients, headaches and symptoms of brain edema were observed during first weeks after reirradiation. All of them received steroids and in majority of them (except two with progression of the disease after SRS), the symptoms subsided. No seizures correlated with SRS were observed, and patients who previously suffered from epilepsy did not require modification of their standard treatment. No anesthetic complications or infections were observed. The majority of patients had no deterioration in performance status (ECOG 0 or 1–82% during FU after SRS compared to 91% before SRS).

**Fig. 1:**
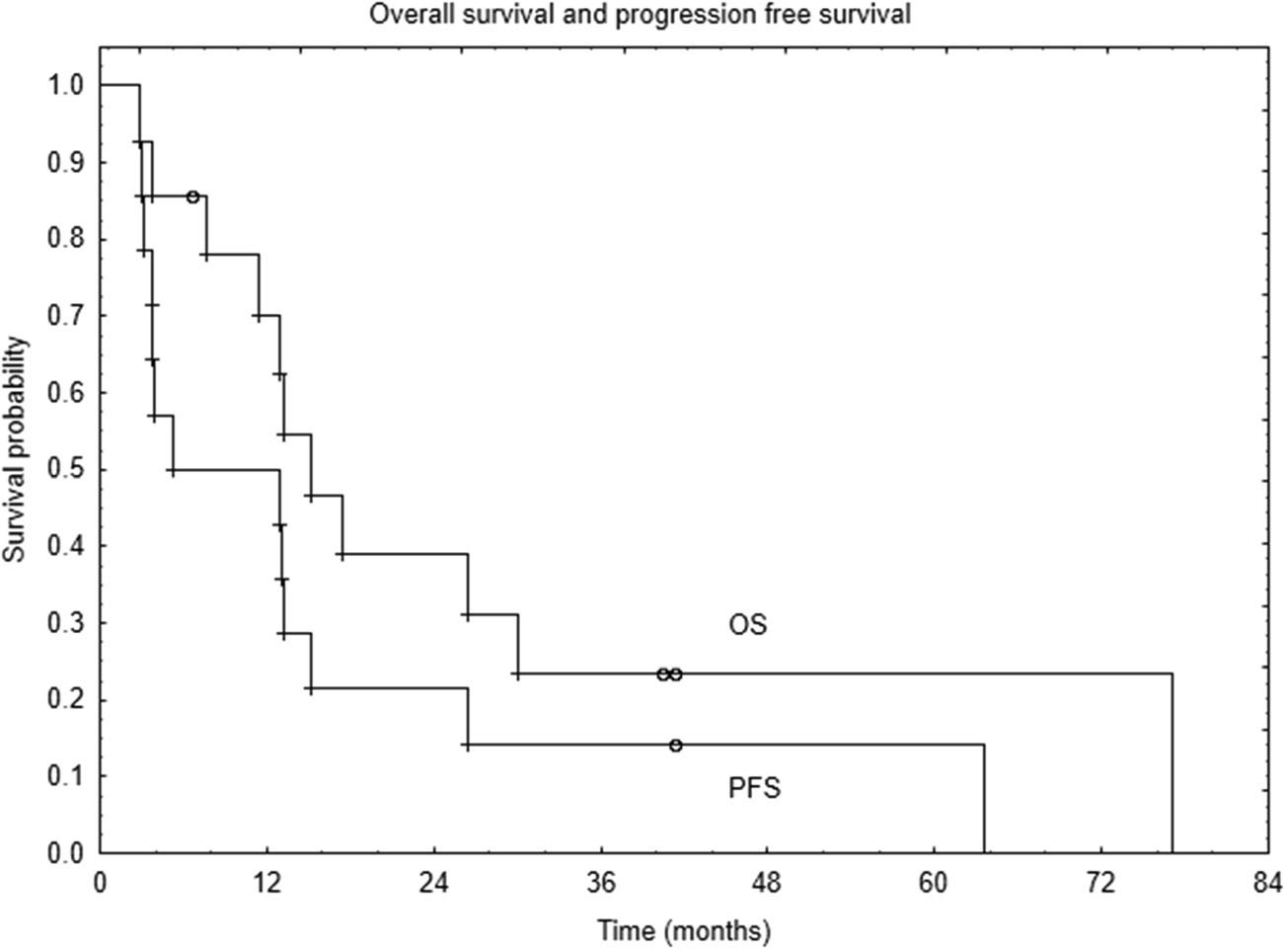
Overall survival and progression-free survival

During follow-up, eight patients had disease progression within or outside the irradiated region (overall disease progression rate was 67%, Fig. 1). Three patients had another surgery, five received systemic treatment, and four received radiotherapy. Fraction dose ranged from 2 to 6 Gy and total dose was within 9 to 18 Gy. One patient received 3 fractions of 3 Gy (total dose, TD 9 Gy), two 3 fractions of 6 Gy (TD 18 Gy), and one was irradiated with fraction dose of 2 Gy to TD of 18 Gy.

During the study period, eleven patients died. The follow-up for the 3 patients still alive was 6.7, 40.5, and 41.4 months, respectively. One- and 2-year overall survival (OS) was 70% and 39% (Fig. 1). Unfortunately, neither National Cancer Registry nor Regional Civil Registry offices provide with the information about the cause of death, and we decided not to call families of the patients due to ethical reasons.

**Fig. 2:**
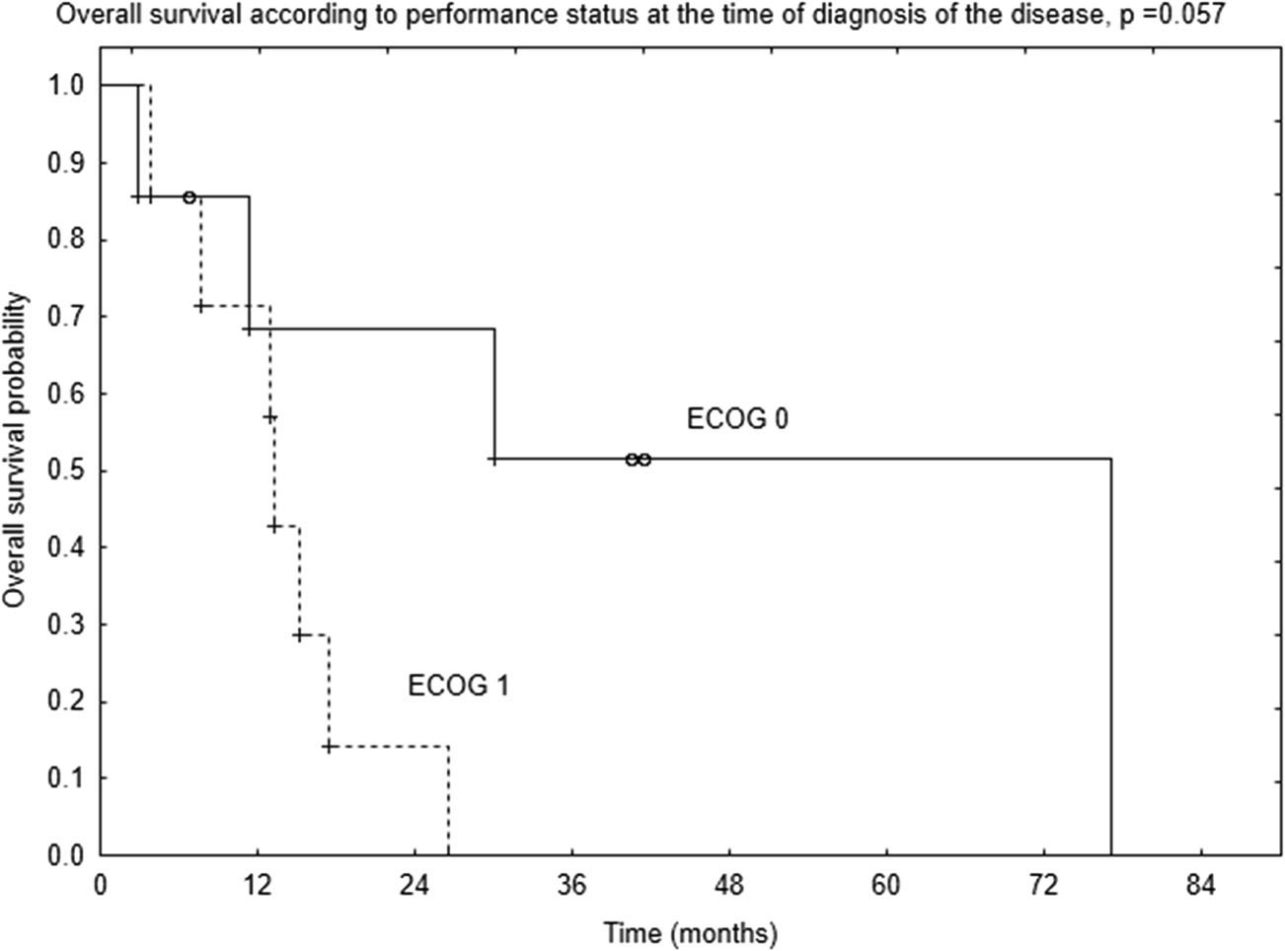
Survival curves according to patients’ performance status at the time of diagnosis of the disease

**Fig. 3:**
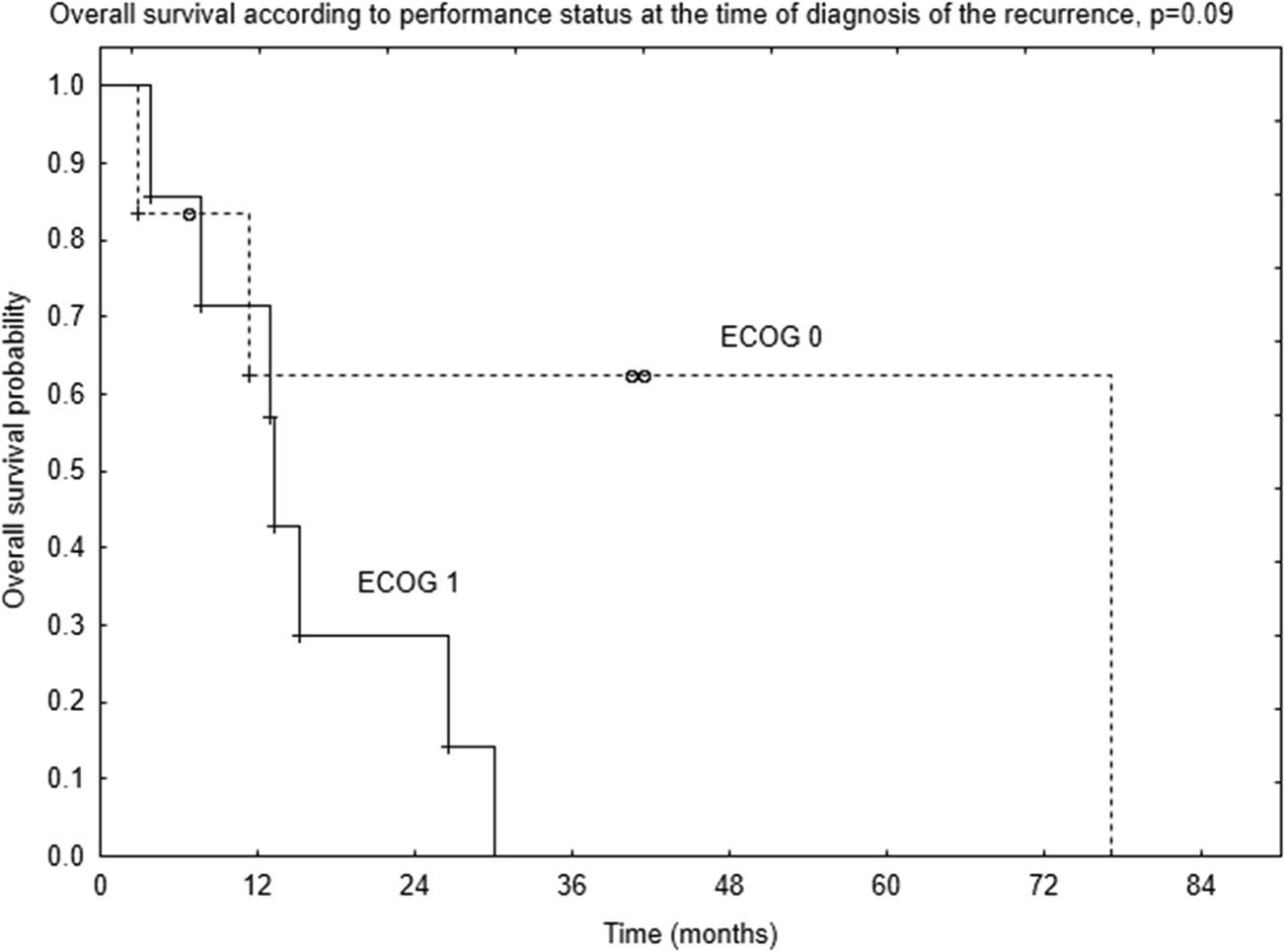
Survival curves according to patients’ performance status at the time of diagnosis of the recurrence

Patients in ECOG performance status of 0 at the time of diagnosis of the disease tended to have better 2-year OS compared to patients in ECOG performance status 1–68% vs. 14% (*p* = 0.057, Fig. 2). Patients in ECOG 0 at the time of diagnosis of the recurrence tended to have better OS compared to those in ECOG 1, but the difference was not statistically significant (*p* = 0.09, Fig. 3).

## Discussion

CNS reirradiation, especially in children, is rarely used due to concerns of its possible toxicity [10, 26–28]. It is not a standard treatment, but in patients with recurrent MB after multimodal therapy (surgery, CSI, CTH), there is a lack of established standards [16]. The results of recent studies on patients with recurrence who received various multidrug chemotherapies, high-dose chemotherapy with bone marrow transplantation combined with surgery, or other treatment options are still poor, and cure is rare [7–9, 16, 22, 23].

The number of publications concerning reirradiation after CSI is small, and the number of patients described in all the reports does not exceed 300 cases [12–25].

Studies on reirradiation of patients who received radiotherapy of cerebrospinal axis as the first-line treatment are shown in Table 3.

**Table 3:** Publications on reirradiation after craniospinal irradiation [10–23, 25]

Study	Number of patients	Doses applied in salvage treatment	5-year overall survival	Toxicity of salvage treatment
Rao AD et al. [25]	67	18.6–70.1 Gy (median 53.1), median fd 1.8 Gy	Median OS MB, 8.4 months; ependymoma, 20.5 months; whole group 5-year OS, 35%	1 case of radionecrosis
Wetmore et al. [16]	38	Mean TD of 38 Gy (range 18–54 Gy)	5-year OS, 55% ± 14%; 10-year OS, 33% ± 16% (from the date of recurrence)	Increased incidence of necrosis
Bakst et al. [15]	13	TD 30 Gy, fd 1.5 Gy	5-year OS 65% (from the date of recurrence), 12 patients received CTH; median OS 37 months	1 case of asymptomatic, in-field necrosis
Saran et al. [17]	14	TD 30–40 Gy in 6–8 fractions	5-year OS, 20%; median OS, 29 months	No toxicity.
Abe et al. [11]	12	Median peripheral TD 20 Gy (normalization on isodose 50%)	2-year OS, 33% ± 14%; 3-year OS, 25% ± 13% (all patients received CTH after SRT, 5 + PBSCT); median OS, 19 months	1 case of brainstem edema, 1 patient died due to toxicity of CTH
Massimino et al. [23]	17	7 patients TD 20.2 Gy, fd 1.3 Gy, 3 patients TD 50 Gy	10/17 received RT, all HD CTH ± PBSCT; for whole group: 5-year OS 20%, median OS 41 months	Not reported for RT
Bauman et al. [10]	14	Not specified for MB	Median OS 11.5 months	No radiation necrosis
Chojnacka et al. [19]	6	TD 40 Gy, fd 2 Gy	Median OS 17.5 months, 1 death during FU (83% alive)	No grade 3–5 toxicity
Milker-Zabel et al. [20]	20	Mean TD 24 Gy (SRT) or 15 Gy (SRS)	6-year OS, 35%; median OS, 73 months	No late toxicity
Patrice et al. [12]	14	Median min. TD 12 Gy	2-year OS 45%, 13 patients received CTH; median OS 10 months	No radiation necrosis
Padovani et al. [22]	5	TD 28 Gy, fd 1.8 Gy	All: concomitant temozolomide, 80% alive after mean FU of 25 months	No neurologic toxicity
Bugulione et al. [13]	1	TD 52.8 Gy, fd 1.2 Gy/twice a day	Alive after 18 months	No radiation necrosis
Keshavarzi et al. [18]	1	TD 14 Gy in 1 fraction	Alive after 12 months	No toxicity
Privitera et al. [21]	1	TD 24 Gy	CTH + bevacizumab died with disease after 35 months	No radiation necrosis
Cieślak et al. [14]	1	TD 45 Gy, fd 1.8 Gy	Alive after 15 months	No toxicity

*CTH* chemotherapy, *fd* fraction dose, *FU* follow-up, *HD* high dose, *min.* minimum, *OS* overall survival, *PBSCT* peripheral blood stem cell transplantation, *RT* radiotherapy, *SRS* stereotactic radiosurgery, *SRT* stereotactic radiotherapy, *TD* total dose

The group of patients treated in our center is small, although comparable to the groups described in the literature. Some patients presented in publications concerning reirradiation of CNS received conventional radiotherapy, and the doses used were within the range of 18 to 70 Gy (1.2–2.0 Gy per fraction). Patients who received radiosurgery or hypofractionated stereotactic radiotherapy were treated with the total dose of 12–24 Gy (radiosurgery) to 24–40 Gy (stereotactic radiotherapy) [12–23]. Total doses used in our hospital were similar to those reported in the literature and were within the range of 6 to 30 Gy (median 15 Gy).

With introduction of new WHO Classification of Central Nervous System Tumors in 2017, primitive neuroectodermal tumors are no longer recognized [29]. The patient with PNET in our series was diagnosed in 2005 and died in 2008, and we were not able to reevaluate histopathologic samples according to the new system. As optimal integration of this newly developed system into clinical care is still a matter of active debate and the purpose of the study was the evaluation of efficacy and toxicity of reirradiation in patients who previously underwent craniospinal irradiation, we included that patient into analysis.

Meta-analysis concerning reradiation of patients with recurrent glial tumors showed that in case of irradiation to the normalized total dose of less than 100 Gy, the risk of radiation necrosis of the brain is very low [30]. The results of radiobiological research on cells of the nervous system suggested that partial repair of radiation damage can occur in the central nervous system. Publications concerning neurotoxicity indicate that factors such as maximum tumor diameter, general condition, dose delivered to the tumor, or use of chemotherapy have an effect on its occurrence [10, 30]. These data suggest that reirradiation may be considered in carefully selected patients without increased risk of complications.

The published reports describe lack or low toxicity of reirradiation and good tolerance of the treatment itself [11–22, 26–28]. Necrosis in the irradiated field, observed by Bakst, is in fact not an undesirable event if area of the necrosis is within the tumor and does not encompass the uninvolved brain [15]. Nevertheless, no patient in our series developed radiation necrosis after radiosurgery, which is consistent with observations of other researches. Results of our study suggesting that patients in better performance status at the time of diagnosis of the disease tended to have better overall survival could be a valuable information of possible outcome.

In the available literature, 5-year OS of patients who underwent reirradiation after CSI ranged from 20 to 65% and median OS is within the range of 10 to 73 months [11–23]. These data are, however, difficult to interpret because some researchers reported their results in relation to the date of recurrence and part in relation to the date of salvage therapy. The described treatment regimens vary widely, like in our series, and some of the patients were given concurrent or adjuvant systemic therapy. The results of our study, i.e., 2-year OS of 39% in comparison to the reported 2-year OS of approximately 25%, showed that some patients might benefit from reirradiation [23]. Also, Dunkel et al. reported an improvement in local control when radiotherapy was added to the treatment of the recurrence [8]. Similar observations could be noticed in our series—more than 70% of patients achieved local control in the irradiated volume.

The patients with MB, especially children, are more likely to receive chemotherapy than patients with ependymoma, and the biology of those two tumors differs. What is more, pediatric oncologists are more willing to give another course of chemotherapy than to refer second radiotherapy. As a result, some of the patients in our series received even up to 3 different types of systemic treatment before salvage radiotherapy. Undoubtedly, with more aggressive and less responsive to the treatment disease, even with very precise SRS, the results will be poor. Whether gaining local control with radiosurgery implemented first and chemotherapy used as an adjuvant treatment would give better results than irradiation after exhaustion of possibilities of systemic treatment remains an open question.

There are several limitations of our study: heterogeneous histopathological diagnoses (MB, AE), lack of histopathologic and molecular feature description, and heterogeneous patient population (children and adults) who received SRS/SRT during the long period of 13 years. Furthermore, the recommendation of treatment of anaplastic ependymoma changed in the last years and CSI is no longer standard of treatment in those patients.

## Conclusions

Stereotactic radiosurgery or hypofractionated stereotactic radiotherapy is an effective treatment method of the local recurrence after CSI. It allows for achieving good local response and can be performed safely in heavily pre-treated patients.
